# Making up numbers in Pano languages: idiosyncratic and unconventional base-free quantification inventories in Amazonia

**DOI:** 10.1098/rstb.2024.0224

**Published:** 2025-10-20

**Authors:** Roberto Zariquiey, Rafael Núñez, Mariana Poblete, Alonso Vásquez

**Affiliations:** ^1^ Chana Field Station for Language Sciences and Interculturality, Pontificia Universidad Católica del Perú, Lima 15088, Peru; ^2^ ETH Zurich, Zurich, Zürich 8092, Switzerland; ^3^ University of California San Diego, La Jolla, CA 92093, USA; ^4^ Leiden University, Leiden, Zuid-Holland 2311 EZ, The Netherlands; ^5^ University of California Santa Barbara, Santa Barbara, CA 93106, USA

**Keywords:** Pano languages, Amazonian languages, numerals, base

## Abstract

Amazonian languages typically exhibit very small numeral systems or lack numerals altogether. Increasing economic and cultural pressures, however, often motivate the emergence of more complex inventories for exact quantification. Headwaters Pano languages from Amazonia historically had two lexical items that can be rendered as the numerals ‘one’ and ‘two’. We argue here, however, that they are not either etymologically or synchronically proper numerals (like the English ones are) and can be better glossed as ‘single/one’ (but also ‘a few’) and ‘pair/two’. For larger quantities (‘three’ to ‘ten’), speakers report idiosyncratic quantifying expressions based on different compositional strategies that recruit the lexical items for ‘single/one’, ‘pair/two’, but also ‘hand’ and, in some cases, other body-part expressions and motion verbs as well. We discuss these idiosyncratic quantifying expressions, showing that they do not present systematic and productive number bases; they exhibit unusual patterns of inter- and intra-speaker variability (i.e. they are poorly conventionalized); and they are rarely used in discourse. Based on these properties, we conclude that these quantifying expressions of Headwaters Pano languages are not numerals proper. We then explore the implications of these salient characteristics for the cross-cultural understanding of quantification and the emergence of numerical systems and the study of anumeric languages in Amazonia.

This article is part of the theme issue ‘A solid base for scaling up: the structure of numeration systems’.

## 1. Introduction

Numbers are conceptual objects that grasp exact quantities and are expressed via numerals—oral, written or gestured signs, among other possibilities of conventionalized representation. Among the many interesting features of Amazonian languages, there is the fact that they exhibit notably limited numeral systems, typically denoting quantities within the range of ‘one’ to about ‘four’ [1–4].^1^ Furthermore, some Amazonian languages, like Pirahã, have been claimed to lack numerals altogether [5,6]. The lack of large numeral systems among Amazonian languages has been the focus of vivid interest in the cognitive sciences that investigate the origin and evolution of number concepts and numeral systems [6–10]. Consistent with the general Amazonian pattern, most languages of the Pano family from Amazonia lack well-established numeral systems beyond lexical items meaning ‘one’ and ‘two’. More recently, diverse cultural exchanges and increasing economic pressures have motivated the borrowing or the emergence of more complex quantifying expressions among people from societies that traditionally operate with limited numeral systems [4,11]. In the case of many Pano languages, this has manifested in the creation of innovative (often lexically complex) quantifying expressions, as well as in an extensive borrowing of Spanish numerals.

In this paper, we study these Pano innovative quantifying expressions used in a numeral-like fashion and evaluate the putative bases that have been claimed to serve as building blocks for their formation. We focus on the quantifying expressions of one particular branch of the Pano family (the so-called Headwaters Pano languages; see §2). We analyse the most salient features of these quantifying expressions regarding their etymology, putative base properties and degrees of conventionality. We do this by means of analyses of linguistic data gathered in the field (for details, see §2) and we propose some terminological refinements that we consider necessary for addressing the questions at stake. We finally contrast our findings with Shipibo-Konibo—a Pano language that, exceptionally, exhibits a well-established decimal numeral system that was largely borrowed from Andean Quechua, possibly during the first half of the eighteenth century [12]. With this study, we intend to make a contribution to numerical cognition research from an Amazonian perspective, setting up fundamental lines for future studies.

The paper is organized as follows. In §2, we give a general description of the Pano language family, providing information on Headwaters Pano languages and Shipibo-Konibo, and briefly describing the fieldwork underlying this article. In §3, we introduce some important terminological clarifications required for the detailed analysis we offer here. In §4, we analyse Pano basic quantifying expressions that somewhat correspond to the English *one*, *two* and *five*, showing that in the Pano languages under study, these terms do not etymologically come from true numerals and are not synchronically numerals like their English counterparts are, since they are not part of a counting sequence. In §5, we analyse higher composite quantifying expressions, focusing on the question of whether or not iterated terms recruited to coin larger ones can be considered as manifestations of a base system. We pay attention to various features related to systematicity, productivity, conventionality and use, to argue that in Headwaters Pano languages there are no bases and indeed, most of the reported forms that refer to exact quantities are not lexicalized forms that can be considered as numerals on the basis of our terminological definitions in §3 (but see the discussion on Shipibo-Konibo in §5c). Finally, in §6, we discuss the implications of these salient characteristics for the cross-cultural understanding of numeral and number concept emergence. We close with some conclusions and provide recommendations for future research from an Amazonian perspective.

## 2. Fieldwork and linguistic background

**Figure 1.**
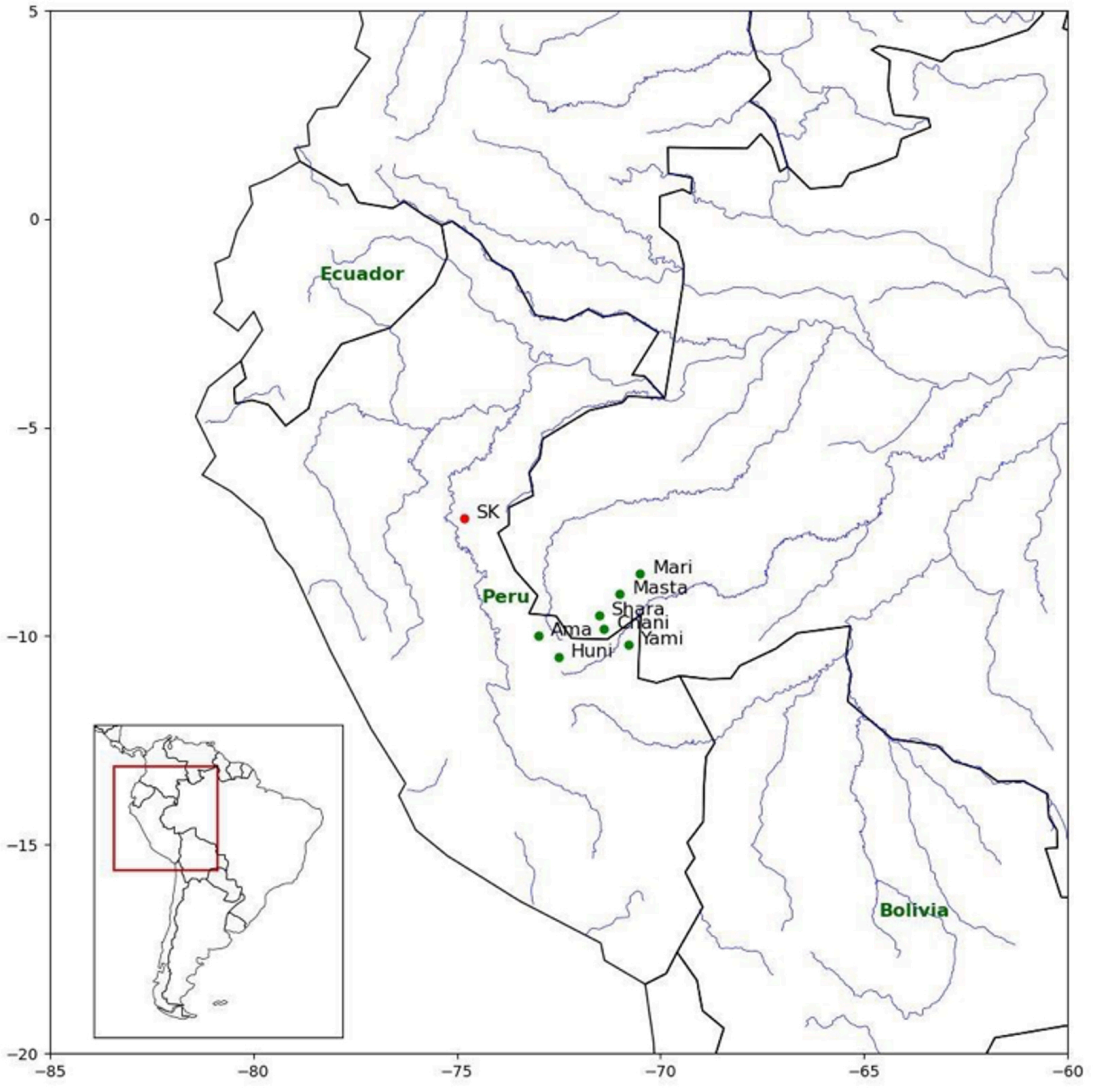
Approximate location of the Pano languages included in this study. Approximate locations of the Headwaters Pano languages are indicated by green dots (Mari = Marinawa, Masta = Mastanawa, Shara = Sharanawa, Chani = Chaninawa, Yami = Yaminawa, Huni = Hunikuin and Ama = Amahuaca). A referential location for Shipibo-Konibo ( = SK) is indicated by a red dot on the map.

Pano is a language family of Western Amazonia comprising some 33 (extant and extinct) languages from neighbouring territories in eastern Peru (Loreto, Ucayali, Huánuco, Madre de Dios regions), western Brazil (states of Amazonas, Acre and Rondônia) and northern Bolivia (departments of Beni and Pando; see figure 1). This article focuses on a cluster of Pano languages mainly spoken along the Yurua and Purus Rivers and their tributaries, often called the Headwaters Pano languages [13]. Headwaters Pano languages constitute a fairly shallow subgroup within the Pano family, and most of them are mutually intelligible.

The data discussed here were gathered during a period of two weeks of linguistic fieldwork conducted in Pucallpa, Peruvian Amazon, in 2023 with the collaboration of speakers of each of seven Headwaters Pano languages: Hunikuin (three speakers), Yaminawa (two speakers), Sharanawa (two speakers), Mastanawa (two speakers), Marinawa (two speakers), Chaninawa (two speakers) and Amahuaca (two speakers).^2^ These observations were made following the ethics protocols established and approved by the Pontificia Universidad Católica del Perú and following the practices established by the Chana Field Station for Language Sciences and Interculturality (https://chana.pucp.edu.pe/).

This fieldwork period focused on the documentation of quantifying expressions and quantity-related concepts, through elicitation (direct interviews with speakers using both verbal and non-verbal stimuli to gather information on quantifying expressions and quantifiers) and *texts*, which is the label used in linguistics to refer to the recording of naturalistic speech (see [14] and [15] for a discussion of the difference between elicitation and texts in linguistic fieldwork). Recorded naturalistic texts focused on the description of different processes related to the elaboration of medicines, food and artefacts, since procedural texts like these are expected to trigger the use of quantifying expressions. The participants ranged in age from 18 to 60, with a relatively balanced representation of genders. Detailed demographic information about the participants is provided in table 1.

**Table 1.** Demographic information of the speakers who participated in this study.

participant code	language	gender	age
Huni_01	Hunikuin	male	52
Huni_02	Hunikuin	female	49
Huni_03	Hunikuin	female	18
Yami_01	Yaminawa	male	48
Yami_02	Yaminawa	male	47
Shara_01	Sharanawa	male	60
Shara_02	Sharanawa	male	35
Masta_01	Mastanawa	female	24
Masta_02	Mastanawa	male	43
Mari_01	Marinawa	female	59
Mari_02	Marinawa	female	27
Chani_01	Chaninawa	female	50
Chani_02	Chaninawa	female	48
Ama_01	Amahuaca	male	43
Ama_02	Amahuaca	female	28

To elicit quantity-related concepts and quantifying expressions, we worked with speakers of each language using pebbles and small sticks in varying quantities. The speakers were asked to express these quantities in their language and to count in both their native language and Spanish. Additionally, we conducted brief interviews to explore whether the speakers believed that counting was feasible in their languages. All interactions were recorded using mobile phones. The fieldwork was conducted by three co-authors of this article, all of whom have expertise in Pano languages, including their lexicon and grammatical structures, as well as prior research experience with speakers of these languages. Lexical elicitation sessions were carried out with speakers of each language working in pairs with two researchers during two separate sessions. This approach ensured accuracy and allowed us to capture any variations in the forms provided by the speakers at different times. Texts were recorded using a video camera, resulting in *ca* 4 h of video recordings, which included both elicitation sessions and recorded texts. Tables S9 and S10 [16] summarize all the quantifying expressions documented during our fieldwork.

## 3. Some terminological remarks

Many human languages have *quantifiers*, which are determiners or pronouns indicative of quantity, like the English terms *all*, *both*, *pair*, *many*, *eight*, *fifty-two*, etc. Some quantifiers are *natural quantifiers* that express quantity in an inexact manner, such as the English terms *several*, *few* and *many*. Others—*exact quantifiers*—express quantity in an exact way, like the form *pair* (which denotes the concept of exactly TWO), *dozen* (which denotes the concept of exactly TWELVE) or the expression *twenty-five* (which denotes the concept of exactly TWENTY-FIVE). While all of these linguistic forms denote exact quantities, only a subgroup of them serves to constitute a counting list to express cardinality, as in the English *one*, *two*, *three*, *four*, etc., and exhibit conventionalized bases for the expression of large quantities. Here, we will refer to these expressions as *numerals*. Thus, terms like *pair*, *dozen* or *half-dozen*, despite referring to exact quantity concepts (TWO, TWELVE and SIX, respectively), will not be considered as numerals, given that in English they are not part of a conventionalized counting list.

When studying the world’s languages from a comparative perspective, this distinction is relevant since, traditionally in linguistic anthropology, it has been unproblematically assumed that all exact quantifiers are necessarily numerals used for counting (e.g. [7,17–20]). This assumption, however, makes important semantic differences between terms like *pair* and *two* indistinguishable—the former referring to a self-contained meaning that does not require the ordered process of counting and that is perceptually readily evoked at once via cognitive subitizing [10], and the latter being part of a counting list and a larger numeral system for which a base can be identified.

In the case of emerging complex quantifying expressions among people from societies that traditionally operate with limited numeral systems, we may also expect to find different degrees of lexicalization, conventionality and systematicity. This may put into question whether or not the implicated forms can be treated as proper quantifiers or numerals (which, since they are linguistic forms, are expected to be conventionalized). For the study of numerals and quantification in languages spoken in under-researched areas, these distinctions are crucial. In this sense, in the present paper, we will refer to the ensemble of linguistic forms used for exact quantification with the more neutral label (*exact*) *quantifying expressions*. The label *quantifying expression* is heuristically more appropriate since it does not claim anything about the participation of the expression in a cardinal counting list (as the term *numeral* does), nor does it assume that the linguistic form under study is a conventionalized/lexicalized item (as the term *quantifier* does).^3^

## 4. On the forms for ‘one’, ‘two’ and ‘five’ in Pano languages: morphologically complex quantifying expressions in the absence of counting

### (a) On the Pano forms for ‘one’ and ‘two’

Pano languages have two lexical items that correspond to some extent to the English numerals *one* and *two*. These Pano words present a high degree of lexicalization and conventionality, and they can be synchronically analysed as basic words. A further look at these forms, however, reveals a diachronic complex internal morphological structure for both. Furthermore, here we demonstrate—to our knowledge for the first time—that etymologically these forms were not originally numerals. Although the etymology of a word is not fully determinant of its synchronic meaning, and not being etymologically a numeral does not preclude a word from becoming a numeral, the discussion here is relevant to understanding the synchronic nature of the forms discussed in this section and their recruitment for the composition of the more complex expressions presented in §5.

For ‘one’, Headwaters Pano languages use the form *ɸɨsti* (with the alternating form *βɨsti*). However, *ɸɨsti* in these languages is not used for counting and, therefore, is not a numeral but rather an exact quantifying expression, as defined in §3. For this reason, we gloss it as ‘one/single’, aiming to reflect its natural usage. In contrast, Shipibo-Konibo employs the related form *βɨstiuʐa* (< *βɨsti + juʐa* ‘body’), which is synchronically closer to the definition of a numeral, as the Shipibo-Konibo people have well-established counting practices based on a decimal system.

Regarding its etymology, the form *ɸɨsti* can be further analysed, as *-ti* is a relatively productive particle meaning ‘quantity’, attested in various Panoan languages. For example, in Yaminawa, *dati* means ‘quantity of this’ (*da* being a proximal demonstrative), and in Shipibo-Konibo, *hawɨti* means ‘how many/much’ (*hawɨ* being an interrogative pronoun). The meaning of the formative *ɸɨs ~ βɨs*, however, is less transparent. We propose an etymology linked to the idea of ‘separated from a larger group’, based on the Shipibo-Konibo verb *βɨsna-* (‘to spread, to scatter’; [21], p. 187) and the Kapanawa verb *βɨstiβo-* (‘to spread’; [22], p. 171).

A significant semantic observation about *ɸɨsti* is that, in natural discourse, it does not always mean exactly ‘one/single’. It is often used to denote small quantities larger than one. While its use to mean ‘one/single’ is conventionalized, *ɸɨsti* also appears to cover meanings such as ‘a few’ or ‘a little’. In Shipibo-Konibo, this distinction is particularly notable: *βɨsti* alone means ‘a few’ or ‘a little’ (21, p. 187) and contrasts with *βɨstiuʐa* (‘one/single’). While *βɨstiuʐa* can also refer to small quantities, *βɨsti* by itself never means ‘one/single’ in Shipibo-Konibo. For instance, in Shipibo-Konibo, one can say both *βɨsti paʐanta mɨniwɨ* (‘give me a few plantains’) and *βɨstiuʐa paʐanta mɨniwɨ* (‘give me one or a few plantains’). The use of *ɸɨsti* as a natural quantifier strongly suggests that it was not originally an exact quantifying expression or a numeral. Similar uses of *ɸɨsti ~ βɨsti* are possible, though less common, in Headwaters Pano languages.

Based on these elements, we propose that the likely etymology of *ɸɨs-ti* is ‘a very small quantity separated from a larger set/group’. Thus, the form *ɸɨsti* for ‘one/single’ was neither originally an exact quantifying expression nor a numeral, as defined in this paper (see §3). The Shipibo-Konibo form *βɨstiuʐa* (‘one’) incorporates the element *juʐa* (‘body’) and can be etymologically interpreted as ‘a body separated from a larger set/group’. As previously noted, *βɨstiuʐa* qualifies as a numeral under the definitions presented here.

Regarding the expressions for ‘two’, the form in all the languages in the sample is *ɾaɸɨ* (*~ ɾaβɨ ~ daβɨ ~ ʐaβɨ*, which are slightly different pronunciations of the same proto-word *ɾaβɨ; see footnote ^3^). Again, among the Headwaters Pano languages, *ɾaɸɨ* is better described as an exact quantifying expression, rather than as a numeral, and therefore we gloss it as ‘two/pair’. The form *ɾaβɨ* in Shipibo-Konibo does satisfy the definition of numeral, since it takes part in a decimal numeral system.

The etymology of *ɾaɸɨ ~ ɾaβɨ ~ daβɨ ~ ʐaβɨ* is very interesting. The form *ɾa- ~da- ~ ʐa-* corresponds to the second syllable of *juɾa ‘body’ and is what is often called a body-part prefix in Pano linguistics (23–26]). The body-part prefix *ɾa- ~da- ~ ʐa-* ‘body’ is combined with the comitative marker *-ɸɨ ~ -βɨ*. Thus, the etymological meaning of *ɾaɸɨ ~ ɾaβɨ ~ ʐaβɨ* is literally ‘with a(nother) body’ and is thus closely related to the idea of ‘pair’ or ‘company’. Again, the term for ‘two/pair’ was not etymologically a numeral, as defined in this paper.

We have shown that the forms for ‘one/single’ and ‘two/pair’ were neither etymologically simple in terms of their morphological structure nor proper numerals. This does not preclude them from having become numerals at some point in their diachronic history, as was indeed the case for Shipibo-Konibo, a language in which they take part in a decimal counting list. Among the Headwaters Pano languages, the forms *ɸisti* and *ɾaɸɨ* are not numerals but exact quantifying expressions that could also be rendered as the English terms *single* and *pair*, respectively, which are terms that, despite quantifying exact quantities, are not used for counting and do not belong to a counting list. Note that *ɸisti* may also mean ‘a few’, which is an important in understanding its diachrony.

### (b) On the Pano forms for ‘five’

Cultural exchanges and increasing economic pressures have motivated the emergence of more complex quantifying expressions among Amazonian languages [4,11], which go beyond ‘one/single’ and ‘two/pair’. Beyond these, speakers of Headwaters Pano languages recruit variable and non-systematic combinations of the linguistic forms for ‘one/single’ and ‘two/pair’, body-part expressions (‘head’, ‘hand’, ‘back’, etc.) and/or motion verbs, as idiosyncratic quantifying expressions. ‘Five’ is expressed via the lexical entry for ‘hand’ in most of these languages. This form is sometimes combined with the form meaning ‘one’ (i.e. ‘one hand’) or with *-ti* ‘quantity’ (i.e. ‘quantity of a hand’), but according to the speakers, these additions are not obligatory. The exceptions are Marinawa, where ‘five’ is literally expressed as ‘two/pair-two/pair-one/single’. Note that in the case of ‘five’, Shipibo-Konibo, which is not part of the Headwaters group, the form for ‘five’ is *pitʃika*, which comes from the Quechua form *pitʃqa* (indeed, this language has borrowed all numerals bigger than ‘two’; see §5c) and is a proper numeral. Table 2 summarizes the terms for ‘five’ in the Pano languages in our sample.

**Table 2.** The forms for ‘five’ in Headwaters Pano languages and Shipibo-Konibo.

subgroup	language	‘five’	morphological structure/etymology
Headwaters	Yaminawa	bɨɸi (ɸɨstiti)	bɨɸi ‘hand’ ɸɨsti ‘one’ -ti ‘quantity’
Headwaters	Chaninawa	bɨɸi (ɸɨstiti)	bɨɸi ‘hand’ ɸɨsti ‘one’ -ti ‘quantity’
Headwaters	Mastanawa	bɨɸi (ɸɨsti)	bɨɸi ‘hand’ ɸɨsti ‘one/single’
Headwaters	Sharanawa	mɨkɨn (ɸɨsti)	mɨkɨn ‘hand’ ɸɨsti ‘one/single’
Headwaters	Marinawa	ɾaɸɨ nun ɾaɸɨ nun ɸɨsti	ɾaɸɨ ‘two/pair’ nun ‘and’ ɾaɸɨ ‘two/pair’ nun ‘and’ ɸɨsti ‘one/single’
Headwaters	Hunikuin	mɨkɨn (βɨsti)	mɨkɨn ‘hand’ βɨsti ‘one/single’
Headwaters	Amahuaca	mɨkɨʃti	mɨkɨn ‘hand’ βɨsti ‘one/single’
Ucayali	Shipibo-Konibo	pitʃika	<pitʃqa (Quechua)

The remaining Pano quantifying expressions come from various combinations of these more basic forms and thus are composite nominal expressions, whose lexicalized (i.e. conventionalized) nature is highly problematic. We analyse these complex forms in §5. Since these combinations may imply the iterative use of certain quantifying expressions, they open an interesting path for studying (the lack of) numerical bases in emerging numeral systems.

## 5. Higher composite quantifying expressions in Headwater Pano languages: are there bases in them?

### (a) Evaluating putative number bases

In linguistics, the label ‘base’ has been used in various ways [27]. Although the term has been borrowed from mathematics, it in turn differs from the original mathematical concept in important ways [28]. In linguistic typology, differences among these multiple characterizations mainly relate to the idea of patterns of compositionality and systematicity in the formation of higher numerals. Fairly general characterizations of bases, such as building blocks for other numerals [29], contrast with more rigorous definitions, according to which a numeral base may be defined as a numerical value that is *systematically* used in the formation of numerals [27]. Note that in the former approach, systematicity is not a central property of numeral bases, whereas in the latter definition, bases are expected to be systematically used in the formation of larger numerals; thus, expressed in modern algebraic notation, a base is the value *n* if numerals are constructed according to the pattern *xn + y,* where *x* is multiplied by the base *n*, to which *y* is added ([30]; see also [3,31]). For example, in *twenty-five*, the base TEN is multiplied by TWO to yield TWENTY, to which FIVE is added. Somewhere in between one finds fully idiosyncratic and non-systematic combinations of quantifying expressions recruited to create higher ones, as well as numeral systems that may have mixed base systems, as when they combine elements from different bases, such as base TWO, base FIVE and base TWENTY [32]. It is worth noting that this linguistic perspective on bases differs from the conceptualization of bases in the study of notations, which places attention on the formal representation and organization of numbers—such as place-value positional systems and specific sets of graphemes—often invoking mathematics to examine how these symbolic frameworks are structured and standardized (e.g. [28]; see also [33,34[].

Regarding the Pano languages from the Headwaters subgroup, it is clear that the quantifying expressions used to create more complex expressions denoting larger quantities do not qualify as bases according to the more rigorous definition of base seen above. Besides, speakers of these languages also often report that their languages have numerals up to ‘ten’ or even ‘twenty’, a claim that has also been made in some published dictionaries. For instance, the Sharanawa dictionary by Scott [35] provides a list of ‘established numbers’ up to ‘twenty’ (table 3). Although the author of this dictionary does mention that ‘the names of the numbers vary among speakers’, he states that ‘most speakers use the names listed [therein]’. Although these languages exhibit fairly lexicalized forms that denote ‘one/single’ and ‘two/pair’ (see §4a), these forms are not recruited in a systematic and conventionalized manner for expressing higher quantities.

**Table 3.** Sharanawa numerals in Scott’s [35] dictionary. The column to the right shows a reproduction of a page from the dictionary with explanations in Spanish. The column to the left presents our English translation.

translation	original
The traditional Sharanahua number system has been replaced by the universal system that they use today. The traditional system was very simple because commercial activity was limited and consisted of two numbers: ‘one’ and ‘two’. The number ‘three’ was formed as ‘one plus two’; the number ‘four’ was ‘two plus two’. For numbers greater than ‘four’, fingers and toes were used: the number ‘five’ was indicated with one hand; ‘ten’ with two hands; fifteen with two hands and one foot; and twenty with two hands and two feet. The names of the numbers vary among speakers, but most speakers use the names listed below. fusti rafu rafu non fusti rafu non rafuri mucu fusti mucu fustiti non fusti mucu fustiti non rafu mucu namancayanoa mucu fustiti non mucu chitushu mucu rafuti tau foshca fusti mucu rafuti non tau foshca rafu tau namancayanoa mucu rafuti non tau chitushu tau fustiti tau fusti non tau foshca fusti tau fusti non tau foshca rafu tau fusti namancayanoa tau fusti non tau chitushu tau rafuti more than 20 ichapa	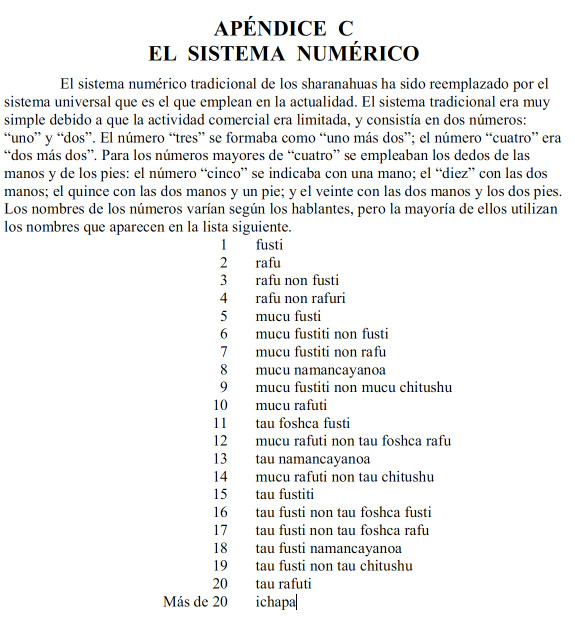

Interestingly, contrary to the claims in Scott’s [35] dictionary, our fieldwork showed that the form of the quantifying expressions other than those referring to ‘one/single’, ‘two/pair’ and ‘five’ varies *substantially* from person to person (and often even the same person provides more than one form). Moreover, based on previous experiences with speakers of all the mentioned languages, we have observed that speakers informally report that terms for numbers above ‘two’ or ‘three’ simply do not exist in their languages and that, for those cases, they just use Spanish terms. During the fieldwork conducted for this paper, speakers were asked to provide numerals up to ‘ten’ or ‘twenty’, and in those cases, we observed interesting patterns of how the quantifying expressions were structured. We observed two strategies for providing quantifying expressions larger than ‘two/pair’. One is based on body-part expressions meaning ‘hand’ (also lexicalized with the meaning ‘five’) and ‘finger’, but also ‘head’ and ‘back’, which are combined in different ways to obtain different quantifying expressions. The second strategy is based on compositionality that combines the basic forms used for ‘one/single’, ‘two/pair’ and ‘five’ in different ways. To express some quantities above ‘five’, alternative quantifying expressions based on these two different strategies were reported by speakers (see electronic supplementary material). Directional verbs were also included by speakers in some of the expressions provided, but their use seems more marginal.

The forms for ‘three’ and ‘four’ are based on the form for ‘two/pair’ by means of combinations such as ‘two/pair and one/single’ and ‘two/pair and two/pair’. Therefore, at least for both these quantifying expressions, the term ‘two/pair’ is iterated in a way similar to a base (with some slight formal differences among speakers; see table 4). Table 4 lists the preferred forms for ‘three’ and ‘four’ in the Pano headwaters languages. Note that the form *-ti* ‘quantity’ is optionally attested in Mastanawa and Sharanawa. The form *nu~nun ~* inun ‘and’ is also optional in all the languages in which it is attested. The form *kimbiʃa* for ‘three’ in Amahuaca is a Quechua loanword, borrowed through Shipibo-Konibo (see §5c). The optionality of the forms -*ti* ‘quantity’ and *nu~nun ~* inun ‘and’ produces a salient formal variability that is dramatically increased in association with numbers higher than ‘five’.

**Table 4.** The preferred forms for ‘three’ and ‘four’ in Headwaters Pano languages. The use of ‘<’ indicates the source language or donor language.

language	three	etymology	four	etymology
Yaminawa	ɾaɸɨ (nu) ɸisti	‘two/pair (and) one/single’	ɾaɸɨ (nu) ɾaɸɨ	‘two/pair (and) two/pair’
Chaninawa	ɾaɸɨ (nu) ɸisti	‘two/pair (and) one/single’	ɾaɸɨ (nu) ɾaɸɨ	‘two/pair (and) two/pair’
Mastanawa	ɾaɸɨ (nu) ɸisti(ti)	‘two/pair (and) one/single’	ɾaɸɨ (nʊ) ɾaɸɨ(ti)	‘two/pair (and) two/pair’
Sharanawa	ɾaɸɨ (nun) ɸisti(ti)	‘two/pair (and) one/single’	ɾaɸɨ (nun) ɾaɸɨ(ti)	‘two/pair (and) two/pair’
Marinawa	ɾaɸɨ (nun) ɸɨstɪ	‘two/pair (and) one/single’	ɾaɸɨ (nun) ɾaɸɨ	‘two/pair (and) two/pair’
Huni Kuin	dabɨ (inu) bɨsti	‘two/pair (and) one/single’	dabɨ (inu) dabɨ	‘two/pair (and) two/pair’
Amahuaca	kimbiʃa	‘three’ (<Shipibo-Konibo)	ɾawɨɾibikɨn ɾawɨ	‘two twos / a pair of pairs’

For expressions denoting quantities larger than ‘five’, we found a substantial amount of variability and diversity. We observed that speakers routinely provided more than one form for the meanings ‘six’, ‘seven’, ‘eight’ and ‘nine’. Some of the forms provided by the speakers recruited body-part expressions associated with ‘hand’, ‘head’, ‘back’ and ‘centre’ (see §4a), whereas others included different combinations of smaller quantifying expressions to refer to larger quantities. In these cases, the form for ‘five’, which is etymologically associated with ‘hand’, was iteratively combined with the forms for ‘one/single’ and ‘two/pair’ to produce ‘six’, ‘seven’, ‘eight’ and ‘nine’ by speakers of all the documented languages in our sample. If the notion of base is applied to these putative numerals, it is ‘five’ that sometimes behaves in a base-like manner since ‘ten’ is expressed as ‘two hands’. In sum, we clearly observe the presence of an alleged base ‘two/pair’ and an alleged base ‘five’ to express the *same* quantity. This raises the interesting question of whether it is adequate to speak of bases in the case of the Headwaters Pano languages’ idiosyncratic quantifying expressions. Are the quantifying expressions ‘two/pair’ and ‘five’ really number bases? The answer depends largely on the role that systematicity, productivity and conventionality play in the definition of 'base'.

#### (i) Systematicity

One central feature of bases is that they are used systematically in a given language. This is what differentiates them from *ad hoc* or idiosyncratic formulations ([27], this issue). In this line, a numeral system is not described as ‘base-5’ if the term for ‘five’ is used in forming only one numeral other than ‘five’. In the innovative quantifying expressions attested in Headwaters Pano languages, neither the terms ‘two/pair’ nor ‘five’ is used in a systematic way. Basically, in most cases, we find a pattern in which the larger basic quantifying expression available is used at the beginning of compositional expressions to refer to quantities. That is, for ‘three’ and ‘four’, speakers use the form for ‘two/pair’, since the form for ‘five’ is not yet available: ‘three’ is expressed as ‘two/pair and one/single’, and ‘four’ is expressed as ‘two/pair and two/pair’. For quantities larger than ‘five’, however, the form associated with this quantity is recruited: ‘six’ is expressed as ‘five and one/single’, ‘seven’ as ‘five and two/pair’, ‘eight’ as ‘five and two/pair and one/single’ and ‘nine’ as ‘five and two/pair and two/pair’ (but note that the combination ‘two/pair and two/pair and two/pair and two/pair’ for ‘eight’ was also attested for Mastanawa as an alternative; see electronic supplementary material); indeed, Marinawa uses quite systematically the element ‘two/pair’ as a base-like element). If the iterated use of ‘two/pair’ and ‘five’ for coining larger numerals is to be taken as the manifestation of a base system, one at best must argue that most Headwaters Pano languages exhibit a mixed base system [32] (Marinawa being an exceptional case since ‘two/pair’ is used more systematically). There are, however, other problems with this analysis, as discussed below.

#### (ii) Productivity

Our field observations show that most speakers were keen to provide quantifying expressions up to ‘ten’ in their languages and argued that beyond that number, one needed to rely on Spanish numerals. For instance, we observed two speakers (Yaminawa and Sharanawa, respectively) who attempted to provide numerals up to ‘twenty’, but with an obvious uncertainty regarding numerals larger than ‘ten’ (note that Scott’s [35] Sharanawa dictionary includes numerals up to ‘twenty’; see table 3). Moreover, even for those speakers, when expressing quantities beyond ‘twenty’, Spanish numerals were in order. In a nutshell, quantifying expressions in Headwaters Pano languages are idiosyncratic combinations of basic forms referring to quantities meant to coin new compositional forms denoting quantities up to ten (or twenty), but they do not appear to constitute a proper numeral system in which a base is used productively to create larger numerals. The attested limitations of the system appear to be specified in part by the fact that the contact language Spanish has a productive decimal system that can readily be extended to express higher quantities. Thus, more rigorous definitions of base ([3,31,36]; see also [27], this issue) will not be easily applied to the quantifying expressions attested among Headwaters Pano languages.

#### (iii) Conventionality

Conventionality is a fundamental property of language and is also a fundamental parameter for identifying proper numerals ([27], this issue). One particularity regarding the numeral systems of Headwaters Pano languages that became obvious during our field observations was the marked variability in the form of the quantifying expressions other than ‘one/single’ and ‘two/pair’. We found both intra- and inter-speaker variation regarding the form of most quantifying expressions. Instances of minimal formal variation relate to the optionality of the forms *nun* ‘and’ and *= ti* ‘quantity’. Basically, in all the quantifying expressions carrying any of those markers in all the languages in the sample, speakers also reported optional forms lacking them. The same happens with the form *ɸɨsti* ‘one/single’, which may or may not appear modifying *bɨɸi* ‘hand’ (*bɨɸi ɸɨsti* ‘one hand’ versus *bɨɸi* ‘hand’). For instance, for the quantifying expression for ‘seven’, all the forms in table 5 were possible and indeed were accepted by speakers of the same language (similar variation patterns were attested for ‘eight’ and ‘nine’).

**Table 5.** The different forms for ‘seven’ attested and accepted by the speakers of the Headwaters Pano languages. They exhibit a radical case of lack of conventionality.

form	**literal translation (expressing ‘seven’)**
βiɸi ɸistɪ nu ɾaɸɨ-ti	‘hand one/single and two/pair-quantity’
βiɸi nu ɾaɸɨ-ti	‘hand and two/pair-quantity’
βiɸi ɸistɪ ɾaɸɨ-ti	‘hand one/single two/pair-quantity’
βiɸi ɾaɸɨ-ti	‘hand two/pair-quantity’
βiɸi ɸistɪ nu ɾaɸɨ	‘hand one/single and two/pair’
βiɸi nu ɾaɸɨ	‘hand and two/pair’
βiɸi ɸistɪ ɾaɸɨ	‘hand one/single two/pair’
βiɸi ɾaɸɨ	‘hand two/pair’

Another type of variability relates to the ‘building blocks’ used for quantifying expressions (particularly for expressing ‘large’ quantities). There is more than one combinatorial possibility for obtaining quantities like ‘eight’ or ‘nine’, and, in some cases, speakers provided more than one possibility to express the same quantity. Attested forms for ‘eight’ in Mastanawa include *bɨɸi ɸɨsti nu bɨɸi ɸɨsti nu ɾaɸɨ* ‘one/single hand and one/single hand and two/pair’ and *ɾaɸɨ nu ɾaɸɨ nu ɾaɸɨ nu ɾaɸɨ* ‘two/pair and two/pair and two/pair and two/pair’. Regarding ‘nine’ in Mastanawa, we encounter *bɨɸi ɸɨsti nu ɸɨsti nu ɾaɸɨ nu ɸɨsti* ‘one/single hand and one/single and two/pair and one/single’ and *bɨɸi ɸɨsti nu ɾaɸɨ nu ɾaɸɨ* ‘one/single hand and two/pair and two/pair’. From the comparison of these forms, it becomes clear that they are not conventionalized lexical items, but rather, they are combinatorial concoctions provided idiosyncratically by speakers to reach a specific quantity.

**Figure 2.**
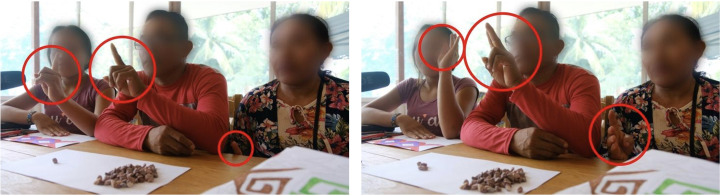
Spontaneous gesture productions exhibited by three speakers of Hunikuin corresponding to the quantifying expressions ‘one/single’ (left) and ‘three’ (right). Note that for ‘one/single’, the two speakers on the left side raise one finger straight up—i.e. the same handshape provided by the profiling of the extended index finger. In sharp contrast, for ‘three’, each one of the speakers displays a different quantity of fingers (four, two and three extended fingers, respectively) produced with radically different handshapes.

Interestingly, we observed that speech–gesture co-production was in accordance with this finding. We noted that basic quantifying expressions for ‘one/single’ and ‘two/pair’, which exhibit null linguistic variability within a given language, are associated with more homogenous gesture productions (i.e. handshapes and placement), whereas larger quantifying expressions triggering linguistic variability also triggered gesture variability—and even gesture incompatibility. Indeed, we noted that some speakers provided numerically mismatched gestures for quantities during elicitation. By ‘numerically mis-matched forms’ we refer to combinatorial hand shapes whose recruited articulators do not add up to the requested quantity (e.g. by gesturing with two or four extended fingers when referring to the quantity ‘three’). This can be observed in figure 2 with the speakers of Hunikuin (similar cases of ‘mismatches’ in speech–gesture co-production were attested in other speakers).

#### (iv) Use

During our field observations, we documented speakers of the Headwaters Pano languages using naturalistic language to describe different activities and processes related to the elaboration of food, medicine and artefacts. We thought this context would be ideal for triggering the use of numeral expressions (as in *add three leaves of this plant*, or *boil three pieces of the bark of this tree*). Our findings, however, contradicted those expectations: Pano quantifying expressions, even the basic ones, were basically non-existent in naturalistic speech. Quantifying expressions in this context, even the basic ones, were always expressed via Spanish numerals, triggering instances of code switching. This is illustrated in the following examples (1) and (2). In these examples, the first line displays the actual uttered expression by the indigenous speaker, which includes the Spanish fragments (appearing in bold). The second line displays the gloss and the third one the approximate translation in English (showing in bold the translation of the code-switched Spanish terms).

(1) nun fechiskiri *casi dos horas*
  we look.for almost two hours
  ‘We look for (leaves) (for) *almost two hours*’
(2) petsakin sharawakin *pa que seca un día*
  group well so (they) dry for one day
  ‘(We) group (the leaves) well so (they) dry for one day’

The fact that the quantifying expressions analysed in the previous sections, which were obtained through elicitation techniques, do not show up in natural texts is telling. This indicates that despite the fluidity of their speech in their native language, Spanish numerals are largely preferred when referring to quantities, which supports the claim that the quantifying expressions in these languages are not in fact truly functional numerals.

### (b) Additional body-based quantifying expressions

For Sharanawa, Hunikuin and Yaminawa, body-part expressions were used in quantifying expressions other than those denoting ‘five’ (literally, ‘one/single hand’) and ‘ten’ (literally ‘two/pair hands’). More precisely, ‘six’, ‘seven’ and ‘eight’ were associated with expressions coined following two different strategies: the strategy described in §5a, exclusively based on the combination of basic quantifying expressions, and another one exclusively supported by body-related items (more specifically, the hands). The numerals with meanings from ‘five’ to ‘ten’ in Hunikuin are presented in table 6. The forms for ‘one/single’ and ‘two/pair’ are presented in table 7 and table 8 for the Headwaters Pano languages and Shipibo-Konibo, respectively. Importantly, none of the attested forms in either table originated as numerals.

**Table 6.** Body-based quantifying expressions in Hunikuin.

numeral	etymology	number
mɨkɨn βɨsti	one/single hand	five
mɨkɨn βɨsti βuʂka	one/single hand (and) a head	six
mɨkɨn βɨsti βuʂka kaʂu	one/single hand (and) a head’s back	seven
mɨkɨn βɨsti namakia	one/single hand (and) (the one) in the centre	eight
mɨkɨn βɨsti mɨkɨn chituʃu	one/single hand (and) the (other) hand’s last one	nine
mɨkɨn dabɨ	two/pair of hands	ten

**Table 7.** The forms for ‘one/single’ in Headwaters Pano languages and in Shipibo-Konibo. None of the attested forms was originally a numeral.

subgroup	language	‘one/single’	morphological structure/etymology
Headwaters	Yaminawa	ɸisti	ɸɨs-ti ‘a very small quantity separated from a larger group’
Headwaters	Chaninawa	ɸisti	ɸɨs-ti ‘a very small quantity separated from a larger group’
Headwaters	Mastanawa	ɸisti	ɸɨs-ti ‘a very small quantity separated from a larger group’
Headwaters	Sharanawa	ɸɨsti	ɸɨs-ti ‘a very small quantity separated from a larger group’
Headwaters	Marinawa	ɸɨsti	ɸɨs-ti ‘a very small quantity separated from a larger group’
Headwaters	Hunikuin	βɨsti	ɸɨs-ti ‘a very small quantity separated from a larger group’
Headwaters	Amahuaca	ti	a shortened version of ɸɨs-ti ‘a very small quantity separated from a larger group’ (ɸɨsti > ti)
Ucayali	Shipibo-Konibo	βɨstiuʐa	ɸɨs-ti + *juʐa* ‘body’ ‘the quantity of a body separated from a larger group’

**Table 8.** The forms for ‘two/pair’ in Headwaters Pano languages and Shipibo-Konibo. None of the attested forms was originally a numeral.

subgroup	language	‘two/pair’	morphological structure/etymology
Headwaters	Yaminawa	ɾaɸɨ	ɾa ‘body’ -ɸɨ ‘comitative’
Headwaters	Chaninawa	ɾaɸɨ	ɾa ‘body’ -ɸɨ ‘comitative’
Headwaters	Mastanawa	ɾaɸɨ	ɾa ‘body’ -ɸɨ ‘comitative’
Headwaters	Sharanawa	ɾaɸɨ	ɾa ‘body’ -ɸɨ ‘comitative’
Headwaters	Marinawa	ɾaɸɨ	ɾa ‘body’ -ɸɨ ‘comitative’
Headwaters	Hunikuin	daβɨ	da ‘body’ -βɨ ‘comitative’
Headwaters	Amahuaca	ɾaβɨ	ɾa ‘body’ -βɨ ‘comitative’
Ucayali	Shipibo-Konibo	ʐaβɨ	ʐa ‘body’ -βɨ ‘comitative’

As table 6, table 7 and table 8 show, body-related quantifying expressions do not operate in terms of number bases. The listings illustrates the remarkable variability and lack of conventionality that we have described so far for Headwaters idiosyncratic quantifying expressions.

### (c) A short note about Shipibo-Konibo

**Figure 3.**
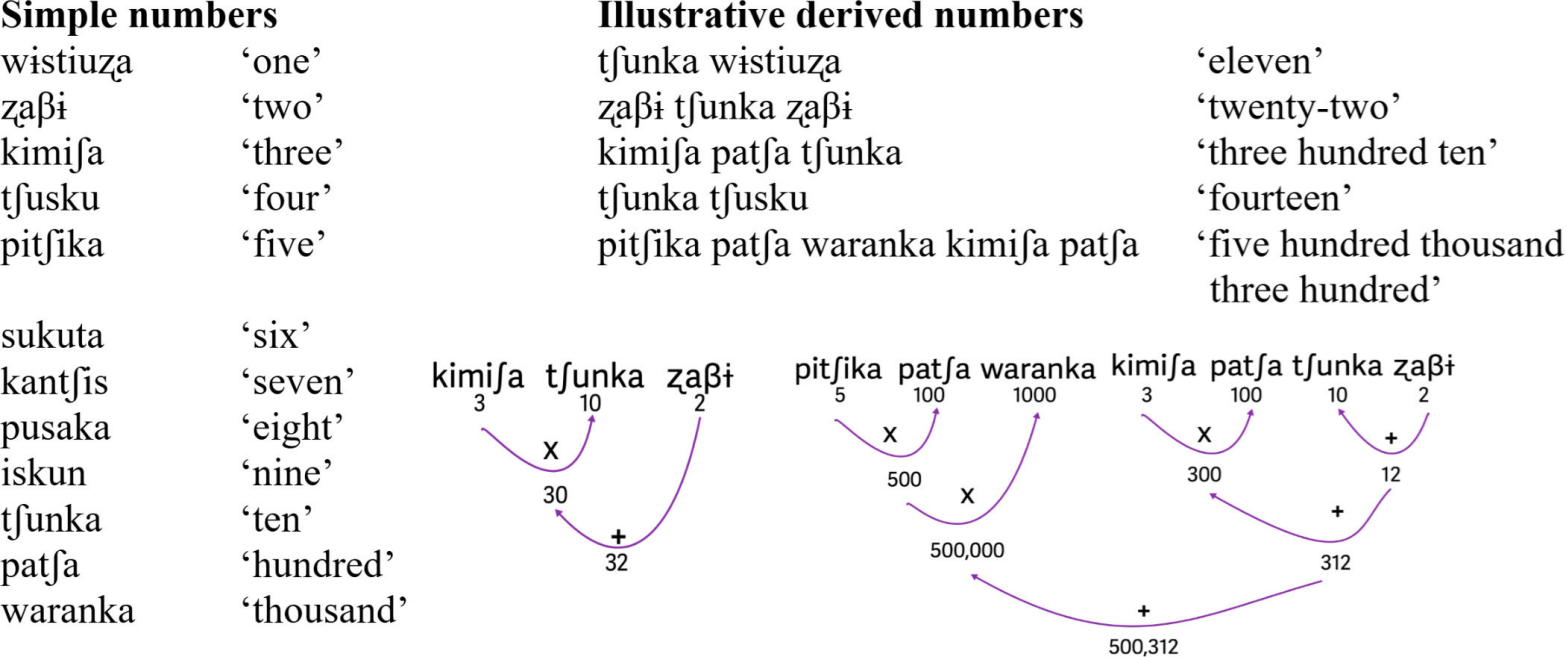
Illustrative examples with explanatory diagram of the decimal system in Shipibo-Konibo.

Shipibo-Konibo is also a Pano language, but it does not belong to the Headwaters subgroup. The relevance of briefly discussing Shipibo-Konibo in the frame of this paper relates to the fact that it has a full decimal system, mostly borrowed from Quechua, likely during colonial times [12]. Shipibo-Konibo numbers are highly conventionalized and widely used among speakers of different ages. Schooling has played a role in the diffusion of Shipibo-Konibo numbers, which are currently largely used by children. The system is fully functional and productive, and the decimal base is systematically attested throughout the inventory. Units follow tens, tens follow hundreds and hundreds follow thousands, very similar to what is observed in decimal systems in Spanish, English and, of course, Quechua (see the examples and the diagram in figure 3). Of course, the skills to produce high numerals and to think of high numbers may vary drastically among speakers. No word for million is attested in the language; speakers report that in that case, they just use the Spanish loan *millón*.

Shipibo-Konibo is a relevant case for this study since it is the only language within the Pano family with a fully productive system—a decimal system—, and therefore it provides an ideal case of comparison for the numeral systems of other Pano languages like the Headwaters languages studied here. It is also relevant from an Amazonian perspective, since most of the languages of the region lack complex numeral systems, and it is also particularly interesting since the donor language is Quechua, another indigenous language—not Spanish or Portuguese—but still external to the Amazonian world. It is important to mention that the Shipibo-Konibo numerals with Quechua origin have been in turn fully or partially borrowed into other Pano languages like Kakataibo [37] and Iskonawa [38] (cf. this is also the case of the form for ‘three’ in the Pano Headwaters language Amahuaca).

## 6. Discussion: what can we learn from Headwaters Pano languages

Amazonian languages typically exhibit very small numeral systems or lack numerals altogether. This raises the questions of how speakers of these languages have conceived and communicated quantification without exact number concepts beyond small quantities. Increasing economic and cultural pressures, however, have been motivating the emergence of more complex quantifying expressions and numeral systems among Amazonian people who traditionally have operated with reduced exact quantifying resources. Epps [11] has investigated the diachronic development of numeral systems in the Nadahup language family of the northwest Amazonian Vaupés region. She has found different situations and stages regarding the evolution and diffusion of numerals in these languages and has showed that even the most basic numerals have transparent etymologies unrelated to abstract number concepts: basic exact quantifying expressions in these languages often derive iconically from concepts related to everyday objects and their etymology is still transparent (e.g. ‘eye-quantity’ to mean ‘two/pair’, or ‘rubber-tree-seed’ that comes in group formations of three distinct seeds), which demonstrates the recent and straightforward nature of their systems of exact quantification. This is exactly the case of Headwaters Pano languages as well. We have shown that not even basic quantifying expressions like the forms for ‘one/single’ and ‘two/pair’ were etymologically numerals. We have shown, indeed, that the form ‘one/single’ in these languages comes from the verb ‘to take apart, or to separate from the rest’. In this sense, the analysis of this form as the numeral ‘one’ is problematic and therefore a better translation for it may be ‘single’ rather than the abstract concept of the number ‘1’. A similar scenario is found for the form for ‘two/pair’ in these languages. We have shown that this form actually means something like ‘with a body or companion’, and it is more accurately associated with the idea of ‘pair’ than to the abstract number ‘2’.

Our findings in Pano languages support the idea that so-called anumeric languages [5–7] were not rare in the Amazonian world. Indeed, they may have been the norm. Although expressions denoting quantity-related concepts can be found in the studied Panoan languages, the evidence shows that they neither appear to be fully conventionalized nor form a systematic counting system in these languages. This suggests that they are recent idiosyncratic innovations. As in Headwaters Pano languages, it may be the case that other languages in the Amazon that currently exhibit numerals innovated them more or less recently as a consequence of social, cultural and economic pressures [11]. This may apply even to basic quantifying expressions, as Pano forms for ‘one/single’, ‘two/pair’ and ‘five’ (which comes from the word for ‘hand’). Thus, the situation described here for Headwaters Pano languages is very similar to what has been described for the Pirahã language and culture [5], for instance.

Beyond basic quantifying expressions, speakers of Headwaters Pano languages provided expressions to denote quantity-related concepts, including ‘three’, ‘four’, ‘six’, ‘seven’, ‘eight’, ‘nine’ and ‘ten’. Such expressions are all morphologically complex and are based on the combination of basic terms or the recruitment of body-part expressions. These quantifying expressions are not productive beyond ‘ten’ or ‘twenty’ and exhibit high inter- and intra-speaker variability. We also observed interesting cases of mismatches between the linguistic production of an exact quantifying expression and its corresponding co-speech gesture production. All this is strong evidence in support of the claim that these quantifying expressions are unstable, poorly lexicalized and conventionalized. In line with this, we showed that these quantifying expressions do not exhibit number bases, as defined in the literature, since the lexical units for ‘one/single’, ‘two/pair’ and ‘five/hand’ are used in disparate and non-systematic manner throughout the range of quantities up to at least ‘ten’ or ‘twenty’. Moreover, these poorly conventionalized and baseless quantifying expressions are rarely (or never) used for counting tasks in natural discourse, involving, for instance, money or the elaboration of complex crafts. Indeed, in cases of discourse recounting manual processes, such as weaving hammocks, building leaf roofs or cooking, speakers of Headwaters Pano languages switch between very few uses of their innovative quantifying expressions and Spanish for quantities up to ‘ten’, and beyond that quantities are systematically expressed by means of Spanish numerals or expressed by other, more general quantifying expressions. While money-related interactions are definitely a result of their recent integration into a global world, recounting traditional crafting processes is something that must certainly predate westernization. Thus, so-called ‘numerals’ in Headwaters Pano languages are indeed not, synchronically, numerals, but rather, they are fairly idiosyncratic forms used to compositionally denote quantities up to ‘ten’ (or ‘twenty’). This is in sharp contrast with the situation in Shipibo-Konibo, another Pano language, which has a fully productive decimal system borrowed from Quechua in the eighteenth century.

The study of quantification systems in the Amazon and their development into numeral systems is still an understudied field. There are over 350 different groups of people in the region, and the literature on empirical studies has primarily focused on a handful of them, such as the Munduruku (e.g.[8,39,40]^8^ and Pirahã (e.g. [6,9]) groups of Brazil. Expanding our knowledge in this field can provide deep insights into the origins of human cognition, language and cultural dynamics. The findings presented here are to some extent preliminary, since more detailed linguistic analyses can be done, covering more languages of the Pano and other families and using rigorous experimental methods. But they are nonetheless particularly interesting and promising in the frame of the discussion about the origin and evolution of numeral expressions and numerical cognition [10]. The questions and issues discussed here need further research. Not only would this help us understand how quantity-related concepts are thought of by speakers of languages that lack complex numeral systems, like the speakers of Headwaters Pano languages, but it would also give us some insight into how exact quantification and number systems emerged in the long and complex human saga.

## Data Availability

Two tables with all the quantifying expression encountered are provided in the electronic supplementary materials. Supplementary material is available online [16].

## Footnotes

^1^
In this article, we will denote numbers by means of words (e.g. ‘twelve’), purposefully avoiding the use of Hindu-Arabic digits (e.g. ‘12’), since the latter require specific written practices and place-value notation—absent in Amazonian practices. The use of words makes the analysis of indigenous quantifying linguistic expressions more transparent. We further adopt the following conventions for the formal treatment of numerals and numerical concepts: specific numerals in Pano languages are written in italics (e.g. βɨstiuʐa in Shipibo-Konibo for ‘one’), while their translations or equivalents in English are enclosed in single quotation marks (e.g. βɨstiuʐa means ‘one’). Universal or abstract numerical concepts appear in upper case without quotation marks (e.g. the concept of ONE). Numerals in interlinear glosses follow the Leipzig Glossing Rules and are written in lowercase (e.g. westiora joni ‘one person’). When numerals are discussed as general linguistic objects, they are also presented in italics (e.g. the numeral one in English). To avoid the confusion triggered by the existence of different orthographic traditions, in this article, we represent Pano forms using representations based on the IPA (International Phonetic Alphabet).

^2^
Sharanawa, Yaminawa, Chaninawa and Mastanawa are fairly mutually intelligible languages.

^3^
For clarity, we must add that there also cases in which speakers use quantifying expressions that are neither numerals nor exact quantifiers but that typically cover a relatively narrow quantity-range and that are typically used in idiosyncratic or *ad hoc* manners (as in the English 'a couple of pairs' meaning ‘approximately four’, or 'nearly a dozen' meaning ‘close to but less than twelve’, and so on).
